# Challenging diagnosis of primary hepatic epithelioid hemangioendothelioma in a patient with familial Mediterranean fever and hypothyroidism: A rare case report

**DOI:** 10.1016/j.heliyon.2024.e38704

**Published:** 2024-09-28

**Authors:** Leen Alarashi, Laila Hamodi, Lamis Abdallah, Mousa Alali, Maher Saifo

**Affiliations:** a Faculty of Medicine, Damascus University, Fayez Mansour Street, P. O. Box: 222, Damascus, Syrian Arab Republic; b Department of Oncology, Albairouni University Hospital, Faculty of Medicine, Damascus University, Harasta M5, Damascus, Syrian Arab Republic

**Keywords:** Epithelioid hemangioendothelioma, Liver neoplasm, Pazopanib, Familial Mediterranean fever, Case report

## Abstract

**Background:**

Epithelioid hemangioendothelioma (EHE) is an extremely rare vascular tumor that accounts for less than 1 % of all vascular tumors. The diagnosis of primary hepatic epithelioid hemangioendothelioma (HEHE) can be challenging because it may be mistaken for other liver lesions. This has led to misdiagnosis in many cases. This study aimed to present a rare case of HEHE that was diagnosed in a female patient with familial Mediterranean fever (FMF) and hypothyroidism who received pazopanib treatment and demonstrated a long-term response to it.

**Case presentation:**

A 43-year-old Syrian woman with FMF who was treated with colchicine for 5 years and hypothyroidism treated with thyroxin was diagnosed with HEHE. She was treated with a tyrosine kinase inhibitor (pazopanib) for over two years and showed a durable response. Although the patient stopped taking colchicine, the episodes of abdominal pain improved after the initiation of pazopanib administration. Pazopanib was well tolerated, and the patient is still alive and doing well.

**Conclusions:**

This study highlights a case of chronic abdominal pain in a patient with multiple comorbidities that resulted in the diagnosis of a rare case of HEHE and prolonged response to pazopanib. In addition, we discuss the effects of pazopanib on FMF symptoms.

## Highlights

• Diagnosing hepatic epithelioid hemangioendothelioma (HEHE) is challenging.
• This is a case of co-occurrence of HEHE and familial Mediterranean fever.
• Pazopanib results in a durable response in hepatic epithelioid hemangioendothelioma.
• Pazopanib may alleviate the symptoms of familial Mediterranean fever.

## 1 Introduction

Epithelioid hemangioendothelioma (EHE) is a unique vascular sarcoma characterized by epithelioid and histiocytoid vascular endothelial cells within myxoid or fibrotic stroma. It can manifest in various parts of the body, including the lungs, bones, soft tissues, and other organs. Hepatic EHE (HEHE) is more common in females than in males. The clinical presentation may include nonspecific abdominal symptoms, with right upper quadrant pain being the most common complaint [1,2].

Differentiating HEHE from other focal liver lesions, such as hepatocellular carcinoma, angiosarcoma, and metastatic carcinoma can be challenging because of its rarity and ambiguous clinical features, which is why a definitive diagnosis relies on histopathological confirmation. The natural course of HEHE varies, ranging from long-term survival to an aggressive course with fatal outcomes. The treatment for HEHE varies from systemic therapy to liver transplantation [1,2].

Familial Mediterranean fever (FMF) is the most common hereditary autoinflammatory disease worldwide. Most affected are populations from the Eastern Mediterranean, including Turks with a prevalence of 0.093 %, Arabs, Armenians, and Iranians. It is caused by mutations in the Mediterranean fever gene (*MEFV*), which has 10 exons and is located on chromosome 16 (16p13.3). The gene encodes pyrin, which plays an important role in innate immunity. FMF is characterized by recurrent, short-lived episodes of peritonitis, usually leading to abdominal pain, reported in >90 percent of patients. Other symptoms include pleuritis, arthritis, and rashes, accompanied by fever. FMF can be diagnosed clinically and based on specific criteria, which can be supported by genetic evidence but not necessarily excluded [3,4].

Since recurrent abdominal pain is an expected finding in FMF patients, the differential diagnosis of abdominal pain in FMF patients may sometimes be inadequate, and the diagnosis of other diseases (such as HEHE) may be difficult. In this study, we aimed to present a unique case of HEHE in a patient with FMF and hypothyroidism who has treated with pazopanib and demonstrated a long-term response. Furthermore, this case highlights the effect of pazopanib on FMF symptoms.

## 2 Case presentation

A 43-year-old unmarried Syrian female patient. She was referred by a surgeon to our cancer center in November 2021 because of a mass in the liver. She is a nonsmoker and does not consume alcohol. The patient was obese, with a body mass index of 60.55 kg/m^2^. She was also diagnosed with hypothyroidism and was being treated with thyroxine beginning in 2015. The patient had no history of oral contraceptive use. The patient had no family history of inflammatory diseases. She reported that she had an uncle with liver cancer and an aunt with gastric cancer.

Since 2010, the patient has been suffering from recurrent abdominal pain in the form of attacks that do not respond to analgesics, accompanied by diarrhea, vomiting, fatigue, fever, and mild joint pain. These attacks increased beginning in 2015 and last for 3–5 days, with a frequency of one attack every two weeks.

Gastrointestinal endoscopy revealed a 1 cm polyp at the pylorus, and biopsies revealed a tubular adenoma with focal high-grade dysplasia, with no neoplastic changes. Abdominal ultrasonography revealed foci consistent with benign hepatic hemangiomas and an adrenal gland adenoma. The patient underwent cholecystectomy and appendectomy in 2016 without any improvement in abdominal pain. On the basis of clinical symptoms of recurrent fever attacks, abdominal pain, joint pain, and fatigue that last for three days or more with a frequency of one attack every two weeks, in addition to laboratory tests that indicate an increase in white blood cells (12,800/mm^3^, neutrophil/lymphocyte: 65/32 %), erythrocyte sedimentation rate (45 mm/hr), and fibrinogen (500 mg/dL) during the attack, a diagnosis of Mediterranean fever was made. She started treatment with colchicine, which contributed to the improvement of her abdominal pain and the decrease in the frequency of attacks to an attack every month and a half, which strengthened the diagnosis (the presence of several major and minor criteria) [5,6].

Owing to obesity, the patient underwent gastric sleeve surgery in July 2021. During surgery, the surgeon noticed masses in the liver and removed one for histopathological examination. The hepatic tissue showed a proliferation of mildly atypical discohesive round epithelioid cells, which presented as scattered intracytoplasmic luminal formations containing red blood cells with a fibrotic background. The immunohistochemical (IHC) study predominantly revealed positivity for CD34 and focal positivity for EMA and CK. It was found to be a low-grade sarcoma, which was consistent with HEHE.

**Fig. 1:**
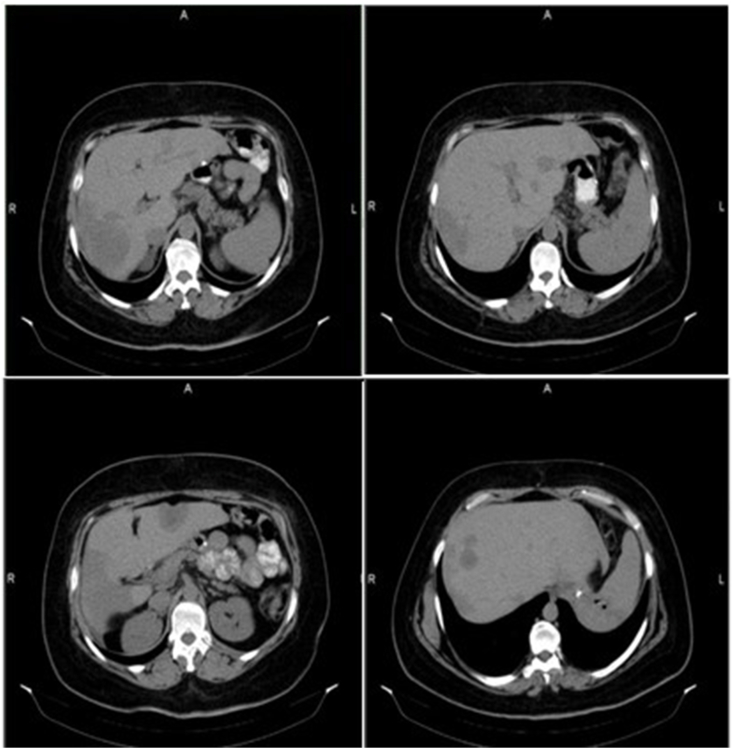
A computed tomography scan at the beginning of diagnosis of a patient with primary hepatic epithelioid hemangioendothelioma. The scan revealed a triangular hypodense lesion in the periphery of the seventh hepatic segment. Multiple other hepatic lesions were also observed, with the largest measuring 4 cm in the anterior segment of the fourth hepatic segment. In addition, there was a right adrenal mass measuring 2.5 cm, which was consistent with a benign adenoma.

A second pathological examination was performed at our center in November 2021, with the same result. Computed tomography (CT) revealed multiple hypodense lesions involving both liver lobes with a mild peripheral flare following injection. The largest mass measured was 4 cm in the hepatic segment IV. There was a small amount of fluid in the pelvis and a benign adenoma in the right adrenal mass (2.5 cm) (Fig. 1).

**Fig. 2:**
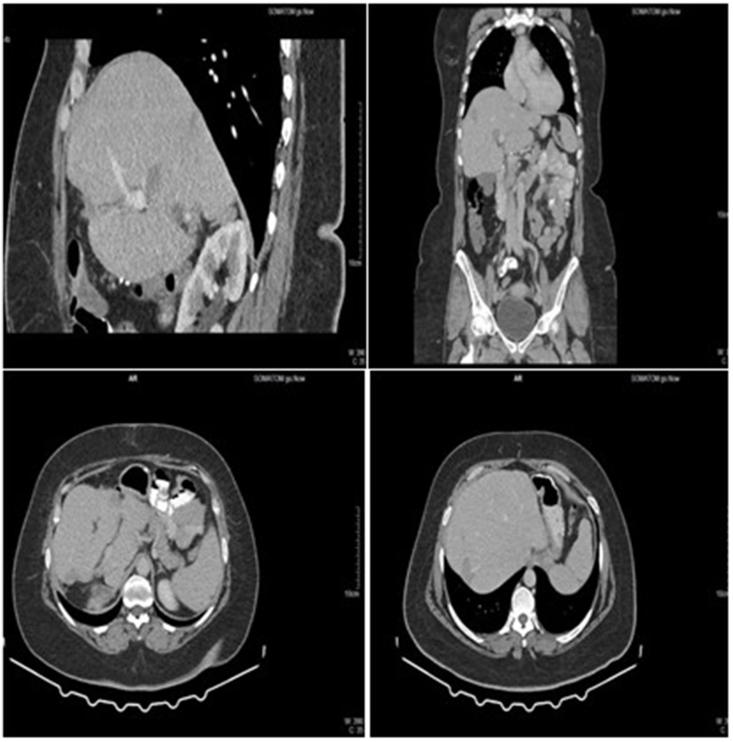
A computed tomography scan after 30 months of initiation of pazopanib revealed a response in a patient with primary hepatic epithelioid hemangioendothelioma. The scan revealed a triangular hypodense lesion in the periphery of the seventh hepatic segment which demonstrated a partial response. In addition, there is a right adrenal mass measuring 2.5 cm, which is consistent with a benign adenoma (stable compared with previous scans).

**Fig. 3:**
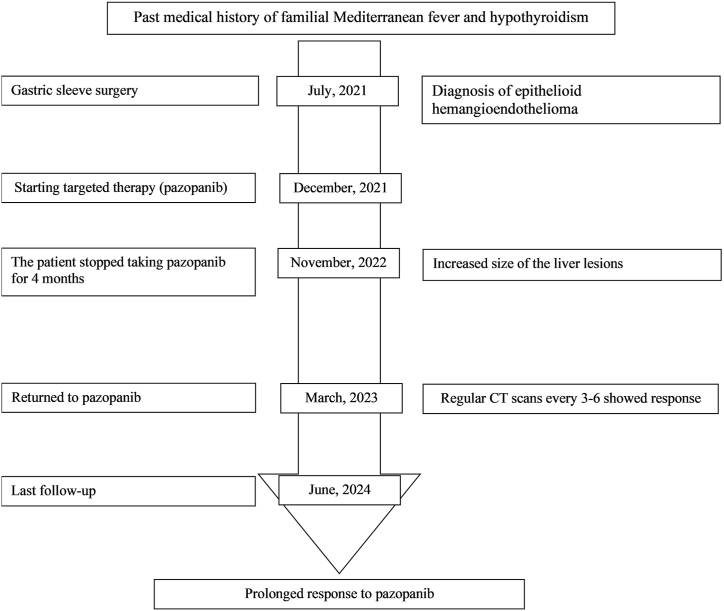
The timeline for a patient with primary hepatic epithelioid hemangioendothelioma.

In December 2021, the patient started pazopanib therapy (800 mg PO qDay), with regular CT scans every 3–6 months. After 11 months (in November 2022), the patient stopped treatment for 4 months, and upon resuming the CT scan, a rapid increase in the size of the masses in the segment IV (3.6 × 6.4 cm) and segment V (18 mm) was noted. Several small focal lesions (6 mm) were observed. In March 2023, the patient returned to take pazopanib with good tolerance, and complete blood count, kidney function tests, thyroid stimulating hormone, and liver function tests were within the normal range. She experienced a feeling of coldness in her legs, mild fatigue, and mild alopecia. She also experienced high blood pressure, which was controlled by diuretics and beta-blockers. Over the next 18 months, the lesions decreased in size (Fig. 2), and the patient's general condition is good. Since the diagnosis of HEHE, the patient stopped taking colchicine. Mild abdominal pain occasionally occurs and often responds to analgesics. A timeline of the patient's case is shown in Fig. 3.

## 3 Discussion

This is the first documented case of HEHE in Syria, and it is also the first case to report the co-occurrence of HEHE, FMF, and hypothyroidism in the literature.

EHE is a rare malignant vascular neoplasm, with an estimated prevalence of less than 1 in 1 million individuals [1,2]. HEHE accounts for approximately 20 % of all EHE cases, and two-thirds are found in women, particularly in middle-aged adults [7].

HEHE is categorized as diffuse, multifocal, or solitary. Twenty-five percent of patients present with nonspecific symptoms, the most common of which are pain in the right upper quadrant, hepatomegaly, and ascites.

The etiology of EHE is not fully understood. Previous cases have been linked to factors such as exposure to asbestos, oral contraceptives, alcohol intake, hepatitis infections, and liver trauma [8]. Although the relationship between FMF and vasculitis has been discussed for many years [6], future studies are necessary to determine the possibility of a relationship between HEHE and FMF or colchicine treatment, as there are no studies on this topic in the literature.

Diagnosing HEHE via clinical, radiological, and pathological methods is challenging. HEHE lesions usually appear hypoechoic on ultrasound. The tumors typically appear as well-defined hypodense lesions on CT, with a peripheral distribution [9]. Approximately 24 % of the lesions presented the target sign, whereas 37.8 % of the lesions presented the target sign with a rim of progressive contrast enhancement [10,11].

Histopathology is currently the definitive method for the diagnosis of HEHE. These tumors can be categorized as moderately cellular or paucicellular and are composed of spindle and epithelial cells that may be present individually or in groups [12]. HEHE demonstrated positive IHC staining for endothelial markers; factor VIII-related antigen was positive in 94 %, and CD31 was positive in 86 % of the tumors [13], and the patient was positive for both CD34 and VIM. Podoplanin (D2-40) was identified as a useful stain for distinguishing HEHE [14].

Molecular testing for HEHE revealed the presence of the *WWTR1-CAMTA1* fusion gene resulting from a t (1; 3) (p36; q25) translocation, confirmed by IHC, demonstrating CAMTA1 expression. A unique subtype of EHE is characterized by the *YAP1-TFE3* fusion gene [15].

Elevated alkaline phosphatase levels are frequently reported to be associated with HEHE; lactate dehydrogenase and aminotransferase may also be elevated. However, in this case, no significant abnormalities were observed in the laboratory values.

The treatment options for HEHE include hepatectomy, liver transplantation, radiation, chemotherapy, and observational follow-up. The highest 5-year survival rate (up to 75 %) has been achieved with hepatectomy. Early diagnosis of the disease is important, as it can be resectable and consequently improve survival. Some individuals may not be suitable for partial hepatectomy because of presence of the multiple lesions. The optimal therapy for patients who do not undergo surgical resection remains unclear. Chemotherapeutic agents such as interferon-alpha, thalidomide, and monoclonal antibodies targeting vascular endothelial growth factor have been documented in HEHE management [1,2,7].

Tyrosine kinase inhibitors, such as sorafenib and pazopanib, have shown efficacy in HEHE. Pazopanib is a multikinase inhibitor that is frequently used to treat renal cell carcinoma and soft tissue sarcoma. Blay et al. [2] reported three patients with EHE treated with pazopanib, with a median progression-free survival (PFS) of 4.6 months. A study by Kollar et al. [16] included 10 patients with EHE treated with pazopanib; the clinical benefit rate was 60 %, and the PFS was 26.3 months. Interestingly, Bally et al. [17] reported the case of a patient with HEHE who demonstrated >8 years of tumor control with pazopanib. Pazopanib was well tolerated, and common side effects of pazopanib include fatigue, diarrhea, hypertension, and bone marrow suppression [17,18]. Patients treated with pazopanib may exhibit abnormalities in liver tests; however, in our patient's case, the laboratory tests were within the normal range.

FMF is generally diagnosed clinically. The classic presentation of symptoms, supported by the presence of a family history and response to colchicine, helps confirm the diagnosis. Laboratory, genetic and radiographic studies may help support the diagnosis or exclude other causes. Many different mutations of the *MEFV* gene have been identified, which could lead to FMF. Among these mutations, the V726A, M680I, E148Q, M694V, and R202Q mutations are responsible for approximately 75 % of cases. Approximately 10 % of patients who are diagnosed clinically with FMF have no mutations in the *MEFV* gene [6]. The decrease in periodic abdominal pain caused by FMF after the discontinuation colchicine in the present study may have occurred because the administration of pazopanib had significant anti-inflammatory effects [19,20]. This effect is also observed after chemotherapy in other autoimmune diseases [21].

The patient in this study had hypothyroidism. Various inflammatory diseases are associated with FMF. In countries where FMF is prevalent, clinicians should understand its co-occurrence to prompt the management of these patients [22]. Furthermore, some reports have shown a relationship between HEHE and the development of hypothyroidism due to increased thyroxine hormone catabolism in the tumor [23]. This may explain the hypothyroidism in the patient in our case.

One limitation of this case is that genetic testing (for the *MEFV* or *WWTR1-CAMTA1* fusion gene) was not performed because it is not available in public hospitals and is not covered by insurance.

## 4 Conclusion

This study presents a rare case of a female patient with HEHE, FMF, and hypothyroidism, with a long-lasting response to pazopanib. This finding highlights the effect of pazopanib on the symptoms of FMF.

## Ethics approval and consent to participate

All procedures involving human participants were performed under the ethical standards of the institutional and/or national research committee and in accordance with the 1964 Helsinki declaration and its later amendments or comparable ethical standards. The study protocol was approved by the Institutional Review Board of Damascus University, and the committee's reference number was MD-140724-278 on July 14, 2024.

## Consent for publication

Written informed consent was obtained from the patient for publication of this case report and accompanying images. A copy of the written consent is available for review by the Editor-in-Chief of the journal.

## Availability of data and materials

The data supporting the findings of this study are available from the corresponding author upon reasonable request**.**

## Funding

This study received no specific grants from any funding agency in the public, commercial, or not-for-profit sectors.

## CRediT authorship contribution statement

**Leen Alarashi:** Writing – review & editing, Writing – original draft, Data curation, Conceptualization. **Laila Hamodi:** Writing – review & editing, Writing – original draft, Data curation, Conceptualization. **Lamis Abdallah:** Writing – review & editing, Writing – original draft, Data curation, Conceptualization. **Mousa Alali:** Writing – review & editing, Writing – original draft, Data curation, Conceptualization. **Maher Saifo:** Writing – review & editing, Writing – original draft, Supervision, Project administration, Conceptualization.

## Declaration of competing interest

The authors declare that they have no known competing financial interests or personal relationships that could have appeared to influence the work reported in this paper.
